# Benchmarking immunoinformatic tools for the analysis of antibody repertoire sequences

**DOI:** 10.1093/bioinformatics/btz845

**Published:** 2019-12-24

**Authors:** Erand Smakaj, Lmar Babrak, Mats Ohlin, Mikhail Shugay, Bryan Briney, Deniz Tosoni, Christopher Galli, Vendi Grobelsek, Igor D’Angelo, Branden Olson, Sai Reddy, Victor Greiff, Johannes Trück, Susanna Marquez, William Lees, Enkelejda Miho

**Affiliations:** 1 Institute of Biomedical Engineering and Medical Informatics, School of Life Sciences, FHNW University of Applied Sciences and Arts Northwestern Switzerland, Muttenz 4132, Switzerland; 2 Department of Immunotechnology, Lund University, Lund 223, Sweden; 3 Center of Life Sciences, Skolkovo Institute of Science and Technology, Moscow 121205, Russia; 4 Department of Immunology and Microbiology, The Scripps Research Institute, La Jolla, CA 92037, USA; 5 Department of Biosystems Science and Engineering, ETH Zurich, Basel 4058, Switzerland; 6 One Amgen Center Drive, Amgen, Inc., Therapeutic Discovery/Molecular Engineering, Thousand Oaks, CA 91320, USA; 7 Computational Biology Program, Fred Hutchinson Cancer Research Center, Seattle, WA 98109, USA; 8 Department of Statistics, University of Washington, Seattle, WA 98195, USA; 9 Department of Immunology, University of Oslo, Oslo 0372, Norway; 10 Paediatric Immunology, Children’s Research Center, University Children's Hospital, University of Zurich, Zurich 8032, Switzerland; 11 Department of Pathology, Yale School of Medicine, New Haven, CT 06511, USA; 12 Department of Biological Sciences and Institute of Structural and Molecular Biology, Birkbeck College, University of London, London WC1E 7HX, UK; 13 aiNET GmbH, Switzerland Innovation Park Basel Area AG, Basel 4057, Switzerland

## Abstract

**Summary:**

Antibody repertoires reveal insights into the biology of the adaptive immune system and empower diagnostics and therapeutics. There are currently multiple tools available for the annotation of antibody sequences. All downstream analyses such as choosing lead drug candidates depend on the correct annotation of these sequences; however, a thorough comparison of the performance of these tools has not been investigated. Here, we benchmark the performance of commonly used immunoinformatic tools, i.e. IMGT/HighV-QUEST, IgBLAST and MiXCR, in terms of reproducibility of annotation output, accuracy and speed using simulated and experimental high-throughput sequencing datasets.

We analyzed changes in IMGT reference germline database in the last 10 years in order to assess the reproducibility of the annotation output. We found that only 73/183 (40%) V, D and J human genes were shared between the reference germline sets used by the tools. We found that the annotation results differed between tools. In terms of alignment accuracy, MiXCR had the highest average frequency of gene mishits, 0.02 mishit frequency and IgBLAST the lowest, 0.004 mishit frequency. Reproducibility in the output of complementarity determining three regions (CDR3 amino acids) ranged from 4.3% to 77.6% with preprocessed data. In addition, run time of the tools was assessed: MiXCR was the fastest tool for number of sequences processed per unit of time. These results indicate that immunoinformatic analyses greatly depend on the choice of bioinformatics tool. Our results support informed decision-making to immunoinformaticians based on repertoire composition and sequencing platforms.

**Availability and implementation:**

All tools utilized in the paper are free for academic use.

**Supplementary information:**

Supplementary data are available at *Bioinformatics* online.

## 1 Introduction

Immunoinformatics has transformed the field of antibody discovery and diagnostics (Brown *et al.*, 2019; Kidd *et al.*, 2014; Miho *et al.*, 2018; Robinson, 2015). It uses computational methods to analyze immunological data, such as antibody repertoire data, and thus leads to a better understanding of the immune system, disease diagnosis, discovery of therapeutics and vaccines (Bock and Goode, 2004; Greiff *et al.*, 2015a; Kidd *et al.*, 2014; Lefranc, 2014; Robinson, 2015; Tomar and De, 2014). Immunoinformatic tools are necessary to process, annotate and classify antibody sequences for a precise, representative and unbiased analysis in order to obtain informative and accurate results.

The adaptive immune system produces a diverse immune repertoire consisting of immunoglobulins expressed on B cells and secreted antibodies, which recognize a variety of antigenic epitopes against a plethora of pathogens. Each antibody is structurally composed of two identical heavy (H) and two identical light chains (L), each chain with a variable and a constant region. Diversity derives from the somatic rearrangement of one variable (V), diversity (D) and junction (J) gene for the heavy chain and a V and J gene for the light chain from a large collection of V, D and J genes present in the germline (Tonegawa, 1983). Additionally, insertions and deletions of nucleotides and somatic hypermutations result in a theoretical diversity of 10^140^ unique antibodies (Miho *et al.*, 2018). The variable region is composed of three complementarity determining regions (CDR1, CDR2 and CDR3), which are flanked by four framework regions (FR1, FR2, FR3 and FR4) that together form the antigen-binding pocket of the antibody. The CDR3 is the most variable region of the antibody and a significant contributor to antigen specificity. Therefore, CDR3 sequence analysis is often an integral component for antibody repertoire analysis in particular for the classification of clones (clonotyping) (D’Angelo *et al.*, 2018; Greiff *et al.*, 2017; VanDyk and Meek, 1992; Xu and Davis, 2000). There are many different definitions of clones in the context of B-cell receptor (BCR) repertoire sequencing in the literature. These definitions can range from identical CDR3 amino acids (a.a.), clusters of similar CDR3 sequences or include the entire variable region (Greiff *et al.*, 2015b; Hershberg and Luning Prak, 2015; Miho *et al.*, 2018; Nouri and Kleinstein, 2018). Unlike T-cell receptors (TCRs), B-cell clonotyping is based on clonal lineages and is usually limited to the heavy chain sequences due to the lesser degree of diversity present in the light chain (Collins *et al.*, 2008; Yaari and Kleinstein, 2015) and because diversity in the CDR3 region of the heavy chain is sufficient for most antibody specificities (Xu and Davis, 2000).

**Fig. 1.**
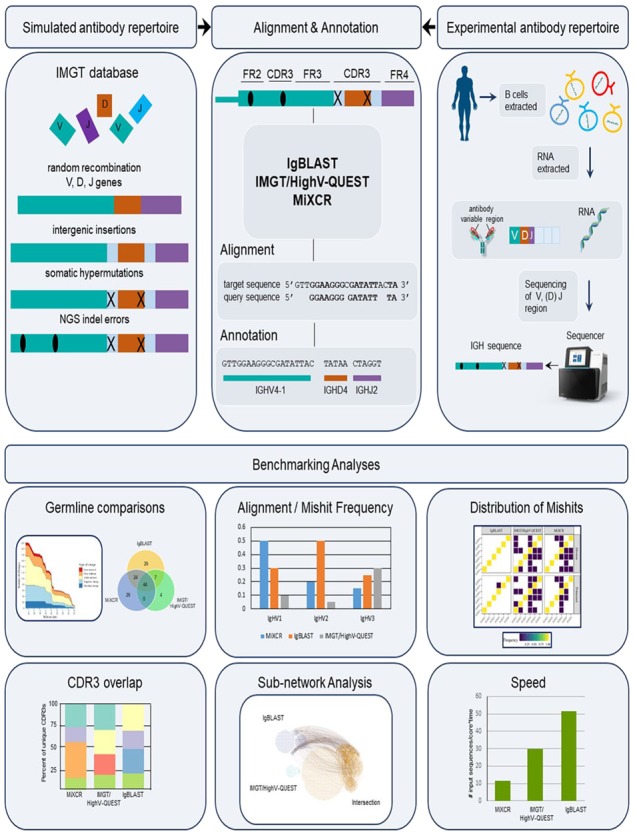
Schematic of immuneoinformatic benchmarking analysis. *Top, Left side*: Antibody repertoire data were simulated *in silico* using IgSimulator. *Right side*: Experimental antibody repertoire data was collected from publically available datasets. *Top, Middle*: Alignment and annotation of simulated and experimental sequences were performed with the immunoinformatic tools: IgBLAST, IMGT/HighV-QUEST and MiXCR. *Bottom*: Downstream analysis of the results compared: (i) the germline databases (ii) alignment (iii) frequency of mishits of the V, D and J gene annotations (iv) unique CDR3 overlap (v) sub-network analysis and (vi) speed of analysis

The general process for antibody repertoire analysis involves the acquisition of immune high-throughput sequencing (HTS) data, filtering, alignment and annotation of the sequences and analysis such as clonotyping (Fig. 1). Briefly, after HTS data are collected, the sequences are aligned to V, (D) and J genes from a reference germline database (e.g.GenBank, IMGT, VBASE2). Sequences are also annotated to define FRs, CDRs and junction sites. Different immunoinformatic tools may provide different options for germline reference databases, alignment algorithms (e.g. Needleman–Wunsch, Smith–Waterman algorithm) and antibody numbering schemes (e.g. Kabat, IMGT, Chothia, Martin), which can result in differences in the analysis (Abhinandan and Martin, 2008; Al-Lazikani *et al.*, 1997; Dondelinger *et al.*, 2018; Kabat *et al.*, 1992; Lefranc, 1997, p. 19).

Although several computational methods are available for the alignment of full-length BCR sequences to germline V, D and J genes and the annotation into FR and CDRs, the benchmarked tools were selected based on their community recognition, available documentation, maintenance cycles, open bugs, similarity between outputs and annotation ability (Supplementary Tables S1 and S2). The performance of these tools has not been compared in terms of accuracy and comprehensiveness with differing germline databases, repertoire compositions and sequencing platforms. Although the alignment and annotation steps are critical to downstream analyses and can dramatically impact subsequent output, it is unknown how the various tools differ and to what extent their features impact the analyses results. Therefore, we set out to investigate alignment and annotation tools for B-cell antibody repertoire analysis. We use *in silico* simulated data (where output results can be linked to known input sequences, originated from defined germlines) and experimental data from three commonly used HTS platforms and specifically investigate differences using six analytical metrics: germline reference databases, percentage of productive output, alignment accuracy to VDJ genes, annotation of CDR3 regions, sub-network analysis and runtime efficiency.

## 2 Materials and methods

### 2.1 *In silico* datasets

Using IgSimulator version 2.0 alpha, we simulated human antibody repertoires by mimicking the biological processes and incorporates artificial error introduced by sequencing (Safonova *et al.*, 2015). Specifically, two immunoglobulin heavy chain datasets set to 1 million sequences each were generated. In order to encompass antibody repertoires of different sequence diversities, two types of repertoire compositions were generated using different parameters. In the simulated diverse antibody repertoire, the number of base antibody sequences was set to 100 000 and the number of mutated antibody sequences was set to 200 000. These parameters generate a high number of low abundant clusters of mutated antibody sequences, which represent a diverse antibody repertoire. For a polarized antibody repertoire, the number of base antibody sequences was set to 20 000 and the number of mutated antibody sequences was set to 100 000. The resulting repertoire consists of a high number of repetitive clusters, which represents a polarized antibody repertoire. The final number of sequences generated by IgSimulator are reported in Supplementary Table S3 and a list of germline genes used by IgSimulator are illustrated in Supplementary Table S5. IgSimulator uses an incorporated default IMGT reference database or a user-defined reference germline (Safonova *et al.*, 2015). Further information on IgSimulator is available in the Supplementary Methods section. The simulated dataset is made available for download as a set for the community to use: https://firebasestorage.googleapis.com/v0/b/aihealthlab.appspot.com/o/public_data%2Fsimulated_sequences.tar.gz?alt=media&token=8ced1886-dd19-40ed-87c7-ee9673bcfa23.

### 2.2 Experimental datasets

Publicly available datasets of human BCR repertoires were selected to represent different sequencing platforms (Illumina MiSeq, Roche 454, Ion Torrent), sample preparation protocols, data quality and disease status (healthy, HIV-infected and meningioma individuals) (Supplementary Table S4). A secondary Illumina MiSeq dataset (B), representative of a modern antibody repertoire sequencing protocols and covers the full V, (D) and J regions was also included.

#### 2.2.1 Illumina MiSeq Dataset A

Peripheral blood mononuclear cells (PBMCs) from a healthy human donor (SRR611538) from the Texas Gulf Coast Regional Blood Center were collected from whole blood and IgG+ memory B cells were isolated (DeKosky *et al.*, 2013). Variable heavy and light chains were paired, amplified and sequenced on the Illumina MiSeq platform.

#### 2.2.2 Illumina MiSeq Dataset B

Peripheral blood was obtained from a healthy individual and B cells were enriched (SRR4026019) (Heiden *et al.*, 2017). RNA was reverse transcribed using biotinlyated oligo dT primer and also incorporated a universal priming site and a unique molecular identifier. PCR reaction was performed using a pool of primers targeting the IGHA, IGHD, IGHE, IGHG, IGHM, IGKC and IGLC regions and against the universal primer resulting in full-length V, (D) and J regions. Illumina specific adapters to added in a secondary PCR. Afterwards, Illumina MiSeq 2 × 300 bp paired-end sequencing was performed.

#### 2.2.4 Roche 454 dataset

PBMCs were obtained from HIV-1-infected donors enrolled in investigational review board-approved clinical protocols at the National Institute of Allergy and Infectious Diseases (SRR924017) (Zhu *et al.*, 2013). RNA was extracted from 5 million PBMCs and reverse transcribed to cDNA. PCR was performed using immunoglobulin gene-specific constant region primers (encoding IgG and IgM) followed by ligation of 454 specific adapters. Resulting libraries were sequenced with the Roche 454 GS FLX pyrosequencing platform.

#### 2.2.5 Ion torrent dataset

Blood was collected from human patients with benign meningiomas and total DNA was extracted (SRR942698). Immunoglobulin specific primers were used to perform a multiplex PCR on the IgH locus and fully rearranged IgH fragments were excised and purified for sequencing by the Ion Torrent PGM sequencer (Gao *et al.*, 2013).

### 2.3 Immunoinformatic tools

Here, we provide a brief summary of each of the immunoinformatic tools. Additional details are provided in the Supplementary Methods section, Table 1 and Supplementary Tables S1 and S2. MiXCR is a bioinformatic tool that processes B- or T-cell immune repertoire data from raw sequences to quantified clonotypes (Bolotin *et al.*, 2015). This involves alignment, annotation and clonotyping. The tool provides PCR error correction and merging of paired-end reads. Analyses were performed using MiXCR version 3.0.5. IgBLAST can analyze both BCR and TCR (Ye *et al.*, 2013). This is done by annotating V, D, J regions, CDR3 identification and mutational analysis of the variable regions using the BLAST search algorithm. The user is required to provide germline databases. IgBLAST v1.12 was used in the analyses. IMGT/HighV-QUEST identifies the V, D and J genes and alleles by alignment with the germline receptor gene and allele sequences of the IMGT germline database (Alamyar *et al.*, 2012; Brochet *et al.*, 2008). This program performs junction analysis and characterizes mutations in the variable region. Analyses reported here were conducted with version 3.4.15. Abstar is a tool that comes as part of the ab[x] package of tools for antibody NGS sequence analysis (Briney and Burton, 2018) and it performs germline gene assignment and primary sequence annotation. We used abstar version 0.3.3 in our analyses. The command lines and parameter descriptions are in Supplementary Table S9.

**Table 1.** Bioinformatic tool scorecard

	IMGT/ HighV-QUEST	IgBLAST	MiXCR
Computational experience	+++	+	+
Reference germline options	+	+++	++
Speed	+	++	+++
Accuracy	++	+++	+
Gene naming granularity	+++ (allele)	+++ (allele)	+ (gene)
Reproducibility with other tools	++	+++	+
Number of unique clones	++	++	+^a^

a MiXCR performs clonotyping which results in lower number of unique clones.

### 2.4 Germline comparisons

IMGT GENE-DB and GenBank provide users with reference germlines for a variety of species and offer different choices of germline reference databases within a species such as including orphon genes, pseudogenes or only using productive genes. IMGT/HighV-QUEST and IgBLAST use the IMGT reference germlines for alignment and annotation (http://www.imgt.org/genedb/) while MiXCR uses a customized database from GenBank. In the following, we specify the options available for each tool, and which germlines were selected. For analysis with IMGT/HighV-QUEST, the default F+ORF+in-frame P was chosen. Using Immcantation repository of scripts, we obtained the IMGT reference germline F+ORF+in-frame P and used it for IgBLAST. The MiXCR built-in reference germline originating from GenBank (repseqio release v1.5) was used. IgSimulator was used with the default IMGT reference database built-in with the tool (reference genes are listed in Supplementary Table S5).

### 2.5 Changes in IMGT germline database

We have extrapolated the release updates for the H*omo sapiens* germline gene reference database from IMGT (http://www.imgt.org/IMGT_vquest/share/textes/datareleases.html) for the past 10 years. We counted and noted the type of change as gene removed from reference, gene added to reference, allele added, sequence changes or metadata changes (when the name or any of the descriptors changes but not the sequence). An interactive version of the germline changes over time for *Homo sapiens* and *Mus musculus* and the underlying data can be found at https://github.com/aihealthlab/imgt-updates.

### 2.6 Alignment and CDR3 overlap analysis

For comparison of gene alignments and CDR3 overlap, IgBLAST, IMGT/HighV-QUEST and MiXCR *annotation outputs* were considered raw outputs. In *preprocessing*, only productive, functional and unique CDR3s are included, and out-of-frame sequences, sequences containing premature stop codons, orphon genes, singletons (CDR3s that only appear once), non-IgH sequences and CDR3s that are smaller or equal to four a.a. were excluded. For IgBLAST and IMGT/HighV-QUEST, the preprocessing is done by filtering the annotations according to the above rules while in MiXCR, clones were exported with additional parameters (-filter-out-of-frames and –filter-stops). In CDR3 overlap analysis, the Top 100 CDR3s are defined as the Top 100 most frequent unique CDR3 a.a. sequences of the preprocessed datasets. Since MiXCR and AbStar CDR3 output differs from IgBLAST and IMGT/HighV-QUEST, we removed the first cysteine (C) and last tryptophan (W) in the CDR3s, in order to make their results comparable to the other tools.

### 2.7 Mishits

A gene alignment is the first gene assigned to an input sequence in the case where multiple assignments are present. A mishit is the alignment of a sequence to an incorrect gene, whereas a hit is the alignment of a sequence to a correct gene (known originating germline). Genes that have identical sequence but differing names are appropriately renamed to avoid false mishits (Supplementary Table S7).

### 2.8 Sub-network analysis

Each annotated dataset was individually expressed as a network where CDR3 sequences are nodes that are connected by a link if they differ by one amino acid (Levenstein distance ≤ 1). The degree of a CDR3 node is defined as the number of connections or links it contains (number of similar CDR3 sequences). From each network, we further analyzed the sub-networks derived from overlapping CDR3 sequences resulting from each tool.

### 2.9 Runtime efficiency

The number of jobs, number of sequences in a dataset and the start and finish times were recorded for each dataset annotation with each tool. For IMGT/HighV-QUEST, each submission is counted as one job.

#### 2.9.1 Instrument

Data were processed on two machines running debian9 with the following specifications: (i) core i7 at 1.8 Ghz with 16 GB memory and (ii) a core i7 at 2.8 Ghz with 12 GB memory.

## 3 Results

### 3.1 Reference germline gene databases change frequently and share few genes

**Fig. 2.**
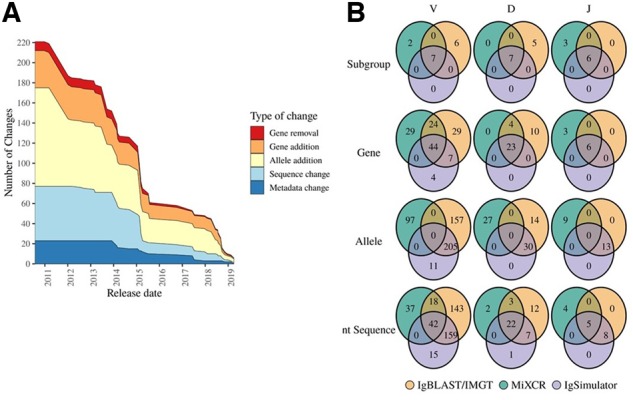
Germline analysis. (**A**) Overall cumulative number and types of changes at each version release date. *X*-axis reports the release date and *Y*-axis represents the number of changes. The different colors indicate the type of change. Red: gene removal—when a gene is removed from the reference. Orange: gene addition—when a new gene subgroup is added. Yellow: allele addition—when a new allele is added. A gene addition here counts as an allele addition with the exception when allele addition is larger e.g. adding multiple alleles for an existing subgroup. Light blue: sequence change—when the nucleotide (nt) sequence of a gene has been updated. Blue: metadata change—when anything except the nucleotide sequence has been changed: e.g. name, functionality, etc. (**B**) Intersection of V, D and J germlines used in the evaluation of immunoinformatic tools separated by gene subgroup name (e.g. IGHV1), gene name (e.g. IGHV1-18), allele name (e.g. IGHV1-18*01) and nucleotide (nt) sequence. The nt Sequence panel measures the number of shared nucleotide sequences, as identical strings, among the tools. For IgBLAST germlines are F+ORF+ in-frame P sequences downloaded with Immcantation scripts. This reference germline was also selected for IMGT/HighV-QUEST. For MiXCR the default germline that is included in the tool was used. IgBLAST/IMGT/HighV-QUEST, orange; MiXCR, green; and IgSimulator, purple

In the first step in antibody repertoire analysis, sequence reads are aligned to a reference database of germline sequences (Fig. 1). Choosing the appropriate reference germline database is of high consequence, as this will determine the genes annotated and affect downstream analyses such as clonotyping. Furthermore, any changes made to the reference germline are also important and can challenge analysis reproducibility. To measure the number and type of changes to the IMGT reference germline, we extrapolated updates made to the *Homo sapiens* reference germline over the last 10 years. Figure 2A shows the cumulative number and type of changes in the reference germline database between two dates. From the first record in IMGT in July 28, 2010 (version 201030-3) to the current germline (February 10, 2019; version 201914-2), a total of 221 changes have occurred: nine gene removals, 37 gene additions, 98 allele additions, 54 sequence changes and 23 metadata changes (Fig. 2A, Supplementary Table S6). More recently, between December 2, 2019 and February 4, 2019, there were six changes: one gene addition, four allele additions, one sequence change (Fig. 2A, Supplementary Table S6). Interestingly, there was a high number of changes prior to 2016, with considerably less alterations to the database thereafter. The large number of changes prior to this date likely reflects the concurrent increase of high-throughput gene sequencing due to technological developments and accessibility to low-cost HTS. The lower number of changes after 2016 may reflect a saturation of reference germline sequencing or IMGT policy changes for gene or allele submissions. The highest percentage of changes during this period was allele additions, which will likely continue as technology costs decrease, sequence quality increases and as sequencing genomes become a commonplace practice. We also found that most of the changes (90 percentile) happened within 32 weeks and introduced at most five changes (Supplementary Fig. S1). The average time update was every 13 weeks with an average of four changes (Supplementary Fig. S1). A dynamic figure of changes introduced to the human and mouse IMGT germline database is shown here: https://aihealthlab.shinyapps.io/imgt-updates-master/. Overall, germline updates seemed to be becoming more frequent, regular and with few changes.

Another confounding factor in antibody repertoire analysis is the tools’ option(s) for reference germlines. IMGT/HighV-QUEST, IgBLAST and IgSimulator all use IMGT-based reference germlines and MiXCR uses GenBank-based germline sequences. Even within IMGT-based reference germlines, the time and choice of reference germlines selected (IMGT contains several germline databases within a single species) can affect annotation results through the inclusions, exclusions and changes to the germline genes themselves. To investigate the differences in the reference germline database between tools, we compared germline gene families (e.g. IGHV1), genes (e.g. IGHV1-2), alleles (e.g. IGHV1-2*01) and nucleotide sequences (e.g. CATTCGT) used by each tool. Of particular note, MiXCR did not contain allele level designations. For IMGT/HighV-QUEST, the default germline reference set was used to reflect an agnostic user’s choice and the same reference germline was implemented for IgBLAST. For IgSimulator and MiXCR, the built-in germline databases were used. A comparison of the gene subgroups, genes, alleles and sequence nucleotides demonstrated that IgSimulator, IgBLAST, MiXCR have 55, 104 and 97 V genes in their respective reference germline databases. All three tools share only 32% (44/137) V genes (Fig. 2B). IgSimulator, IgBLAST and MiXCR have 4, 29 and 29 (≈ 3%, 21% and 21%) V genes that are tool-specific and are not included in the other tools’ reference germlines (Fig. 2B). This demonstrated that the choice of a particular reference germline can influence results by the inclusion or exclusion of certain genes or alleles. Interestingly, MiXCR did not provide information of the allele (corresponding MiXCR zero overlap because of this reason), which can limit this tools’ application for genetic research (Fig. 2B). We also found sequence differences among genes represented with the same name (Fig. 2B). Considering that there are differences within the reference germlines, if certain genes are important for analysis, the researcher should be aware of the contents of each germline as they can influence repertoire analysis results. These germlines also change frequently so vigilance and awareness are necessary as this may affect the reproducibility of results.

### 3.2 Alignments of sequences to V, D and J genes differ across immunoinformatic tools

**Fig. 3.**
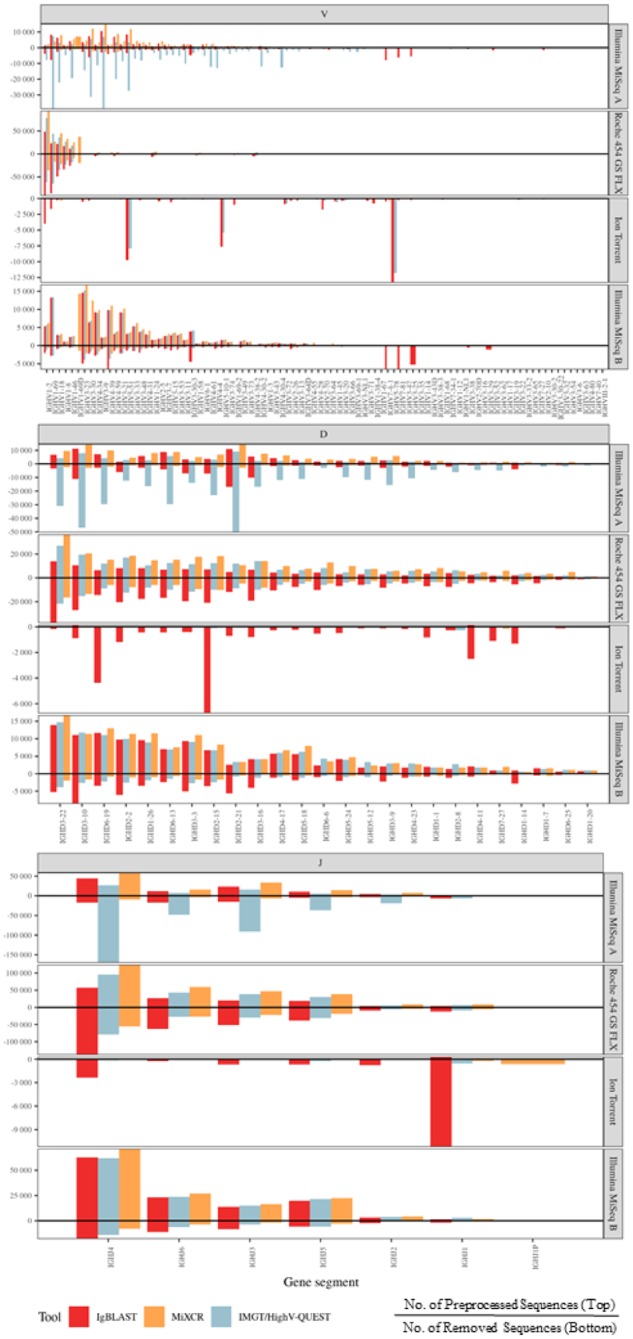
Alignment counts for each tool with experimental datasets. We compare the ability of each tool to align a given sequence to a particular gene (V, D and J). The hit counts before and after preprocessing and across different platforms (Illumina MiSeq, Roche 454 GS FLX and Ion Torrent) are shown. Preprocessing is defined as the removal of unproductive sequences (out-of-frame sequences, sequences containing premature stop codons, orphon genes or non-IgH sequences). Sequences that are removed by preprocessing by each tool are expressed by negative counts on the bottom while remaining preprocessed data is expressed on the top by positive counts. IgBLAST, red; MiXCR, orange; and IMGT/HighV-QUEST, blue. Only data for genes with total annotated count above the 30th percentile is shown

Next, we compared the ability of each tool to align a given sequence to a particular gene (V, D and J) and investigate the impact on the results before and after preprocessing and across different sequencing platforms. Next, we compared the ability of each tool to align a given sequence to a particular gene (V, D and J) and investigate the impact on the results before and after preprocessing and across different sequencing platforms (Fig. 3). We found that while IMGT/HighV-QUEST aligned the most sequences of the V genes in the Illumina Miseq Dataset A (450 569 sequences) compared to MiXCR (155 774 sequences) and IgBLAST (200 164 sequences), IMGT/HighV-QUEST also lost a significant portion (86.9%) by preprocessing compared to MiXCR (16.3%) and IgBLAST (52.8%) (Fig. 3). This trend is also true for D and J gene annotations but does not persist for annotation results from the MiSeq dataset B, Roche 454 or Ion Torrent machines. With the MiSeq Dataset B, which is more in align with modern sample preparation techniques, 37% of total V genes aligned by IgBlast were filtered out (lost) by preprocessing, while IMGT/HighV-QUEST lost 20.6% and MiXCR lost 10.7% which was similar to the Roche 454 dataset results. This trend persisted for D and J genes for both datasets (Fig. 3). IgBLAST was the only tool that was able to significantly align Ion Torrent V gene data but most of this data (99.3%) was filtered out by preprocessing: leaving only 0.7% of annotated reads after preprocessing (Fig. 3). MiXCR and IMGT/HighV-QUEST were only able to align a small number of sequences in the Ion Torrent dataset. These patterns are consistent with D and J gene alignments for the Ion Torrent sequencing data (Fig. 3). This may be likely due to the low number of sequences in this dataset as well as the quality of data. Looking at individual V, D and J genes it is evidence that there are differences between gene alignment results between the three tools. Overall, our results show that each tool aligns the same sequences differently, and that alignment results also depend on the dataset quality and sequencing platform.

### 3.3 MiXCR and IMGT/HighV-QUEST have the highest mishit frequencies

**Fig. 4.**
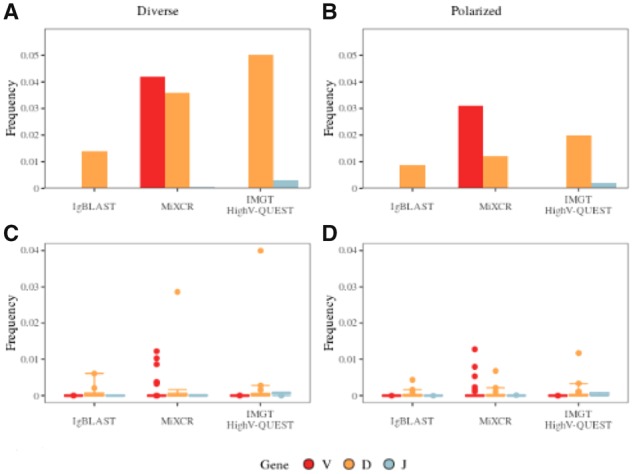
Frequency of annotation mishits. Top: These figures show the cumulative frequency of annotation mishits of each gene (V, red; D, orange; and J, blue) per each tool with simulated (**A**) diverse and (**B**) polarized datasets. Frequency is calculated by dividing the number of annotation mishits (K) divided by the number of total annotations (N) by the tool. Frequency = K/N. Bottom: The boxplot panel shows the frequency of annotation mishits per subgroup (e.g. IGHV1) within each gene for simulated (**C**) diverse and (**D**) polarized datasets. This is calculated by dividing by the number of mishits of a particular subgroup (W) by the total number of hits within that subgroup (X). Frequency = W/X. The lower and upper hinges correspond to the first and third quartiles (the 25th and 75th percentiles)

The accuracy of gene annotation by the tools was assessed using simulated human IGH sequences with differing compositions (polarized and diverse). Since MiXCR does not annotate at the allele level, the analysis was done at the gene level. We found that that there are several gene names and allele names that contain the same nucleotide sequence, of which immunoinformaticians should be aware and are listed in Supplementary Table S7. In conjunction with a given tool’s preference for one gene name over another, this creates further complexity in evaluating accuracy. We found that MiXCR had the highest frequency of V gene mishits (0.042 and 0.031) for diverse and polarized simulated repertoire compositions. IMGT/HighV-QUEST had the highest frequency of mishits for D (0.05) and J (0.002) genes for diverse, and similarly for polarized (D, 0.020; J, 0.002) simulated dataset although at lower frequencies (Fig. 4A and B). Overall, MiXCR contains higher frequencies of mishits across repertoire compositions and genes (average frequency of mishits, 0.020) while IgBLAST has the least overall frequency of mishits (average frequency of mishits, 0.004).

To determine if errors in gene annotations are uniformly distributed across all genes or are biased to a particular group of genes, we analyzed the distribution of error within gene families. Surprisingly, we found that the medians were quite low or at zero but the average frequencies were skewed higher due to outliers in V and D genes for each tool (Fig. 4C and D). The frequencies of J genes did not have any outliers (Fig. 4C and D).

To further investigate which genes were the outliers, we generated heat maps to characterize misidentified genes within each repertoire composition. We found that each tool had different genes that were misidentified within the same dataset (diverse or polarized) (Supplementary Fig. S2). There were also differences in frequency of incorrect annotations of the same tool with differing repertoire compositions with the diverse repertoire having more mishits than the polarized dataset (Supplementary Fig. S2). Interestingly, V gene annotations seemed to have the least number of genes misidentified among the three tools than D or J gene segments (Supplementary Fig. S2). MiXCR had the high frequency of incorrectly annotating IGHV3-66 to IGHV3-53 at a frequency of 0.49 for diverse and 0.76 for polarized while IMGT/HighV-QUEST and IgBLAST annotated them correctly 100% of the time in both repertoires (Supplementary Fig. S2). The alignment of these two genes is shown in Supplementary Figure S3. This pattern is also true for gene IGHV4-4 which only correctly aligned at a frequency of 0.28 in diverse and 0.33 in polarized datasets with the exception of IgBLAST in the diverse dataset which incorrectly aligned IGHV4-4 at a frequency of 0.003 (Supplementary Fig. S2).

In addition, IGHD7-27, a very short gene, had a lower frequency (0.25 and 0.40) of correct alignment using IMGT/HighV-QUEST (high mishit) compared to MiXCR (0.46 and 0.63) and IgBLAST (0.89 and 0.76) in the diverse and polarized simulated datasets (Supplementary Fig. S2). The small size of this gene may affect how each tool is able to annotate it.

For J gene annotations, none of the tools exceeded a mishit frequency greater than 0.01 but there was a different distribution of misidentified genes across the different tools (Supplementary Fig. S2). Although accuracy is a difficult metric to calculate based on the duplication of gene sequences and the small size of some genes, we attempted to assess how each tool performed with the same simulated datasets and found that IgBLAST had on average the most accurate annotations.

### 3.4 Wide range of unique CDR3 a.a. output overlap among tools in annotated data, after preprocessing and Top 100 CDR3s

Although each tool finds similar unique annotated output counts, there is a wide range of unique CDR3 sequences shared among the tools that varies depending on dataset and data type (e.g. annotated, preprocessed or Top 100). In annotated output data, overlap ranges from 10.4% to 31.9%, in preprocessed data from 4.3% to 77.6% and within the Top 100 most frequent CDR3 sequences it ranges from 31.9% to 77.5% between datasets.

Of these shared sequences, MiXCR in general has the highest percentage of CDR3 sequences that are found by the other two tools in annotated datasets (MiXCR, 21.4%; IgBLAST, 17%; and IMGT/HighV-QUEST, 18.8%), but in preprocessed data IgBLAST is found to have the highest percentage on average (68%). There were two exceptions in the diverse dataset and the Illumina MiSeq B dataset where IMGT/HighV-QUEST has the highest percentage of overlap (of immunoinformatic). On average, the two tools that provide the most similar CDR3 sequence outputs were IgBLAST and IMGT/HighV-QUEST which have an overlap of 30.7% in annotated data, 12% for preprocessed and 33% for Top 100. Output from different tools resulted in the most overlap when looking at the different datasets individually. This can be observed in the diverse, preprocessed data and the Roche 454 annotated and preprocessed data where MiXCR and IMGT/HighV-QUEST had the most overlap. IgBLAST and MiXCR consistently had the least amount of overlap.

**Fig. 5.**
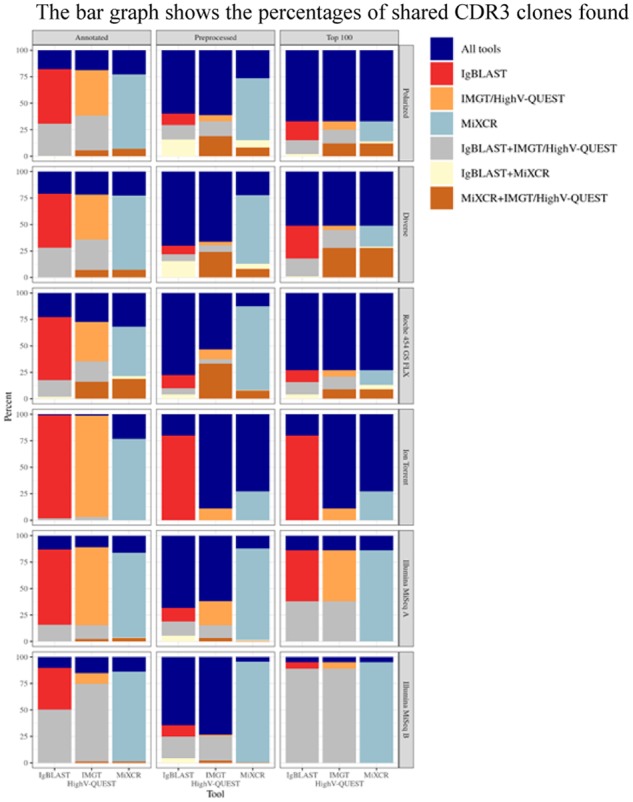
Overlap of unique CDR3s. The bar graph shows the percentages of shared CDR3 clones found by each tool as annotated outputs, preprocessed and the Top 100 frequency-based clones for each simulated and experimental dataset. Preprocessed sequences are sequences that contain no premature stop codons, out-of-frame sequences, CDR3 sequences are longer than five amino acids, and contain only unique CDR3s. Percentages were calculated by dividing the number of unique CDR3s found or shared between tools by the total number of CDR3s found by that particular tool. Blue, all tools; red, IgBLAST; orange, IMGT/HighV-QUEST; light teal, MiXCR; gray, IgBLAST and IMGT/HighV-QUEST; yellow, IgBLAST and MiXCR; brown, MiXCR IMGT/HighV-QUEST

The tool that had the highest number of unique CDR3 sequences that did not overlap with results from other tools was MiXCR in annotated (70.5%), preprocessed (76.8%) and Top 100 CDR3s (41.7%) with IgBLAST having lowest non-overlapping results at (54.4%, 11%, and 32.3%, respectively) (Fig. 5). In order to benchmark resulting CDR3s, we used the tool abstar to annotate the datasets. Abstar annotated a high number of unique CDR3s compared to the other tools (data not shown) but CDR3 overlap remained low.

To investigate the cause in CDR3 overlap disparity between tools, IMGT/HighV-QUEST and IgBLAST alignments and annotations were compared. As they both used the same germline gene sets, we can begin to quantify algorithm and alignment differences. For IgBLAST, 6.82% (51 791/759 505) of total number of input sequences did not have overlapping CDR3s with IMGT/HighV-QUEST and 1.95% (14 844/759 505) for IMGT/HighV-QUEST (Supplementary Table S8). Of these input sequences, the 1% (7735/759 505) had the same VDJ gene segment alignment but different CDR3 annotations suggesting the tools algorithms are likely responsible for this difference (Supplementary Table S8). The contribution of input sequences that had different VDJ gene segment alignments resulting in different CDR3 annotations was 0.16% (1248/759 505) (Supplementary Table S8). This suggests that this is due to different alignment processes by each tool. This analysis suggests that alignment and annotation in addition to germline gene sets are contributing factors to the disparity between CDR3 a.a. overlap.

### 3.5 Sub-network analysis

**Fig. 6.**
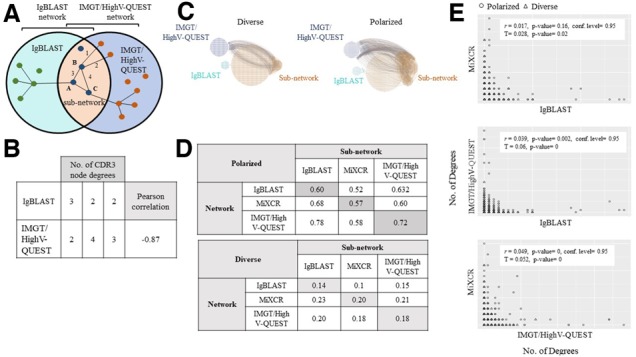
Sub-network analysis. (**A**) Schematic of a network comparing CDR3 sequences (black dots = nodes) similar to each other (connected by links = lines) by one a.a. and found by each tool exclusively or both (sub-network). (**B**) Example of how CDR3 node degrees are calculated on the subnetworks and example of Pearson correlation between the two tools’ degrees. (**C**) Example of a sub-network generated by the comparison of two tools (IgBLAST and IMGT/HighV-QUEST) using diverse and polarized simulated datasets (**D**) The shaded gray boxes represent the average degree of the entire network for a single tool. Sub-networks may have different average degree depending on which annotation network the degree is measured: different tools annotated CDR3s result in different degrees for the same sub-network. Rows represent the network where the degree is measured in and the columns represent the sub-network formed by CDR3 shared in the network indicated by the corresponding column name. (**E**) The scatterplot shows the degree value comparison by tool. For each sub-network, we test the Pearson coefficient correlation (*r*) and Kendall rank correlation coefficient (τ) of the CDR3 degrees

To further benchmark each of the immunoinformatic tools, we utilized *sui generis* sub-network analysis to measure the amount or degree of similarity between the CDR3 a.a. sequences annotated by each tool using simulated datasets. A network is built for each tool using the preprocessed CDR3 a.a. sequences where nodes (CDR3 a.a.) are connected by links if they differ by one amino acid. The degree of a CDR3 node is the number of links (or number of CDR3s) that differ by one amino acid. We investigate the sub-networks formed by CDR3 a.a. which are also present in other networks, meaning the CDR3s that overlap between tools. An example of how degrees are calculated and how networks and sub-networks are formed can be found in Figure 6A and B. The networks for polarized and diverse CDR3 of annotations from IgBLAST and IMGT/HighV-QUEST and their corresponding sub-networks are shown in Figure 6C. To further characterize the differences in annotations between the tools, the average degree of the entire networks resulted from the annotation with each tool were compared (example of this calculation is shown in Fig. 6A and B). The annotation networks follow the expected topology (average number of degrees) for both polarized and diverse repertoires (i.e. when average degree is < 0.5 = diverse and > 0.5 = polarized). This is observed for all tools (Fig. 6C and D). This indicates that our sub-network analysis reflects the properties of the simulated repertoires and thus is a useful analysis to benchmark the output of the tools.

The sub-networks of overlapping CDR3s between annotation networks, can be differentially examined to infer which tool has a higher repertoire diversity based on average degree in the corresponding sub-network (Fig. 6D). The average degree is only changed if the links are not shared between the annotation networks. When comparing the average degrees of IgBLAST’s sub-networks with MiXCR and IMGT/HighV-QUEST (Fig. 6D, first row) to the average degrees of the other tools’ networks (second and third row), IgBLAST has lower degree averages suggesting that IgBLAST has the most diverse sub-networks (it on average has less connections or links with similar CDR sequence nodes), IMGT/High V-QUEST second, with close diversity to IgBLAST and MiXCR last with the least diverse sub-networks (Fig. 6D). Although MiXCR is shown to be the least diverse sub-network, this is likely due to the clonotyping step necessary to identify CDR3s, which the other tools do not perform. As shown previously in Figure 5, the sub-network analysis also demonstrates the differences in annotation between the tools and how datasets’ properties such as diversity can change depending on which tool is used.

For each sub-network, we also tested for correlation between CDR3 degrees of different tools using Pearson’s product moment correlation coefficient (*r*) and Kendall’s tau (τ) to investigate the rank correlation of the degrees. The Pearson correlations (*r*) and Kendall correlations (τ) for all comparisons of the tools were near 0 indicating that the tools generate CDR3 repertoires that result in networks with degree with differing connectivity (i.e. their degree and degree ranks are not correlated among tools) (Fig. 6E). This further demonstrates that the tools result in disparate annotation results.

### 3.7 Runtime efficiency varies between tools

**Fig. 7.**
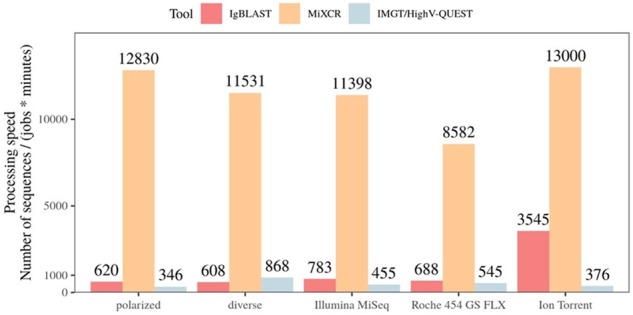
Processing time. The processing speed of each tool with different datasets was measured. The processing speed is defined as: number of sequences processed divided by minutes multiplied by number of computing jobs used. The processing speed numbers are shown on top of each column. For IMGT/HighV-QUEST one job is considered one submission and for IgBLAST and MiXCR jobs represent number of cores used, in order to account for multi-core personal computers. Processing speed = nr sequences processed/(minutes * jobs)

To ascertain the computation times for each tool, the user time for annotation was measured for the three tools using the same input files at varying sizes and using different numbers of jobs (Section 2). For IMGT/HighV-QUEST one job is considered one submission and for IgBLAST and MiXCR jobs represent number of cores in order to account for multi-core personal computers. We calculate IMGT/HighV-QUEST as one job per submission and assume. As shown in Figure 7, MiXCR was the fastest to annotate all datasets while IMGT/HighV-QUEST and IgBLAST finished the jobs at different speeds depending on data type. This figure can also be used to calculate analysis time for any user for these tools if the number of cores and number of sequences is known.

## 4 Discussion

The different immunoinformatic workflows used for antibody repertoire analysis can lead to potentially different results. Therefore, their use does not only impact numerous scientific results reported in peer-reviewed articles (Shah *et al.*, 2019), but also conclusions for diagnostics, immunotherapy, antibody discovery and vaccine design (Lanzavecchia *et al.*, 2016; Maecker *et al.*, 2012; Parola *et al.*, 2018; Trotta *et al.*, 2018). Therefore, analyzing antibody sequencing data accurately and reproducibly is of utmost importance.

Identifying genes involved in immunoglobulin (Ig) rearrangement is highly important in autoimmune and malignant diseases such as leukemia, lymphoma and rheumatoid arthritis where the antibody repertoire can be used for classification and diagnosis (Maecker *et al.*, 2012; Robinson, 2015). IMGT/HighV-QUEST and IgBLAST use the IMGT germline database while MiXCR uses a specific GenBank database for germline sequences. However, IgBLAST and MiXCR can also include other germline databases as well, while IMGT/HighV-QUEST is limited to the IMGT germlines. These different databases contain different set of VDJ genes, which can affect all downstream results as shown in the analysis. Most novel analysis tools are compared to standard tools but there has not been a cross-tool and result-drive benchmarking of the most commonly used tools as of date for antibody sequencing analysis (Bolotin *et al.*, 2015; Ye *et al.*, 2013). The AIRR community is currently working towards developing appropriate metadata fields for documenting novel germline alleles, identifying germline genes not previously documented by IMGT, and establishing standards for databases incorporating IMGT, IgPdb and Vbase (Breden *et al.*, 2017; Rubelt *et al.*, 2017; Ohlin *et al.*, 2019; Vander Heiden *et al.*, 2018). Additionally, there is a need for standardized sample preparations, library construction protocols, validated germline databases and datasets for benchmarking. This effort has been started with the Stanford S22 dataset but actual germline sequences are missing while a reference exists for germline gene names (Jackson *et al.*, 2010).

We found that there is substantial difference between the results from various immunoinformatic tools, which adds to the known PCR and sequencing bias and errors. In this study, we compared three, regularly-used immunoinformatic tools for antibody sequencing data analysis (IMGT/HighV-QUEST, IgBLAST and MiXCR) using simulated and experimental datasets to inform users to the different tools available, default parameters and options, and on the differences in analysis and research results (Table 1, Supplementary Tables S1 and S2). First in the analysis, each antibody sequence is aligned to all possible germline genes contained in a germline database (IMGT and GenBank) using various algorithms. Due to the different database options (IMGT and GenBank, IgPdb and Vbase germline sequences) and restrictions imposed by the tools, there is a discrepancy between the analysis tools is the database of germlines chosen by each of the tools. We also have developed a novel method of benchmarking tools using sub-network analysis for a statistical quantification of the differences between immunoinformatic tools, which may be useful for benchmarking other tools. Of particular surprise was the wide range of in CDR3 overlap between the three tools especially in the Top 100 most frequent CDR3s as this is often the focus on many researchers and is often used in choosing candidates of antigen-specific antibody sequences (Reddy *et al.*, 2010). Based on our analysis, we believe this is a result of confounding factors such as germline gene selection, differences in algorithm, and definitions (CDR3 parameters, unproductive/productive). A more thorough and detailed examination is necessary to further define the differences in CDR3 annotation.

Variables of which users need to be aware of prior to choosing an immunoinformatic tool are: computational experience of user, reference germline, time, accuracy of tool, sequencer, repertoire composition and number of unique CDR3s identified. Novices in immunoinformatics may greatly benefit from the ease of use in IMGT/HighV-QUEST. If speed is an important factor, then MiXCR would be a more appropriate choice. IgBLAST would be best if accuracy is of utmost importance such as in genetic recombination studies. In addition, if alleles are necessary IgBLAST and IMGT/HighV-QUEST would be better choices although an allele-level benchmarking of these two tools would be necessary. For specific sequencer-generated data, it is important to consider the quality of data over type of sequences as repertoire composition does not seem to have a huge effect on misidentifications compared to the tool or reference germline chosen given our small size. As shown in our data, the different tools found differing CDR3s that overlap in different amounts. IgBLAST would be an ideal tool for reproducibility with other tools while MiXCR would be better suited for detecting unique CDR3 sequences not found by other tools. In general, our data suggest that antibody sequencing analysis differ significantly depending on the analysis tool used and there is no single tool that is appropriate for all datasets and a summary of our findings can be found in Table 1. We also provide a list of all currently available tools for any type of BCR and TCR analysis (Supplementary Table S9). In future, studies need to be designed that allow to appropriately assess different forms of bias—the use of biological controls (synthetic sequences) is an option.

Although there are many B-cell repertoire analysis tools, they often contain many shortfalls. While reviewing other immunoinformatic tools, we had to face several challenges such as installation problems, bugs in the software, lack of standard output file types or lack/out of date documentation. The upkeep and maintenance of immunoinformatic tools are very important for their continued use. For the exploring the changes to the IMGT reference germline, we developed an interactive version of germline changes for human (Homo sapiens) and mouse (Mus musculus) which can be found at https://aihealthlab.shinyapps.io/imgt-updates-master/.

Data quality is also very important to consider as low-quality data results can be misrepresented leading to erroneous results. This must be taken into account when using IgBLAST and IMGT/HighV-QUEST as these tools do not provide a statistical summary on the number of reads that were annotated unlike MiXCR.

Metadata for simulated datasets is unavailable because current fields are tailored to biological sequences only. Further work is currently being done in this respect from the AIRR community (https://www.antibodysociety.org/the-airr-community/) in order to set a metadata standard that describes synthetic immune repertoire data originating from simulations.

## 5 Conclusion

We provide a comprehensive overview and comparison of the three most-commonly used immunoinformatic tools, IMGT/HighV-QUEST, IgBLAST and MiXCR for antibody repertoire analysis. We examined and compared reference germlines over time and examined the performance in terms of annotation output differences, accuracy and speed using two simulated repertoires of differing compositions and experimental datasets produced from different sequencing platforms. Additionally, we provide guidance to novice and experienced immune-informaticians on commonly used immunoinformatic tools. Our analysis results show that there is a need for a future cohesion of immunoinformatic tools, analysis pipelines and standards (Breden *et al.*, 2017; Ohlin *et al.*, 2019; Rubelt *et al.*, 2017; Vander Heiden *et al.*, 2018).

## Supplementary Material

btz845_Supplementary_Data
