# Supplementary material for: Benchmarking immunoinformatic tools for the analysis of antibody repertoire sequences
Source: Bioinformatics. 2019 Dec 24;36(6):1731–9. doi: 10.1093/bioinformatics/btz845 (PMC7075533; doi:10.1093/bioinformatics/btz845)
Supplement: btz845_Supplementary_Data [file btz845_supplementary_data.docx]

**Supplementary Information**

**Supplemental Methods**

*In silico* datasets

IgSimulator. The IgSimulator tool simulates human antibody repertoires by mimicking the biological processes and incorporates artificial error introduced by sequencing (Safonova et al., 2015). Briefly, IgSimulator recombines randomly selected V, (D), and J genes and includes intergenic nucleotide insertions at junction sites to generate sequences that mimic antibody repertoires. The resulting sequences are called base antibody sequences. The frequency of each base antibody sequence is assigned using the power-law distribution where each frequency represents the number of times each base antibody sequence will be present in the repertoire. Next, IgSimulator introduces somatic hypermutations (substitutions, deletions and insertions) into each base antibody sequence (throughout the variable region but in higher frequency in the CDR regions) generating mutated antibody sequences and then again randomly assigns the frequency of each mutated antibody sequence using power-law distribution. The resulting antibody repertoire is further subjected to ART in order to mimic the error rate of a NGS Illumina sequencer MiSeq operated using the 2x250 bp chemistry (Huang et al., 2012). Several user-determined parameters influence the composition of the repertoire: number of base antibody sequences, number of mutated antibody sequences and repertoire size. To simulate a diverse or biologically naïve antibody repertoire, (1) a large number of base antibody sequences and (2) a small difference between number of mutated antibody sequences and number of base antibody sequences is required. To generate a polarized or immune challenged antibody repertoire, similar to what one would expect to find in a class-switched, affinity matured repertoire, (1) a smaller number of base antibody sequences and (2) a larger difference between number of mutated and number of base antibody sequences are selected.

Immunoinformatic tools

MiXCR. It uses a version of the k-mer based KAligner for alignment to reference germlines (Liao *et al.*, 2013). The tool aligns V, D, J and constant (C) regions to a built-in default germline sequences custom generated from GenBank nuccore records of genome assembly (IGH, NG_001019.5), performs clonotyping and is able to rescue low quality reads by mapping them to previously assembled high quality clonotypes, increasing its quantitative capabilities. MiXCR only annotates to the gene level (e.g. IGHV1-2) and not the allele level (e.g. IGHV1-2*02). MiXCR uses a Sequence TreeMap for error correction and the IMGT numbering scheme for region delineations. It also uses a built-in library of reference germline V, D, J, and C gene sequences for human, mouse, and rat. It is also possible to import germlines from any species from IMGT library. The repseq.io tool (https://github.com/repseqio/repseqio) contains the built-in reference germline and allows for the import of user-defined references.

IgBlast. IgBLAST was developed at the National Center for Biotechnology Information (NCBI) to facilitate antibody sequence analysis. IgBLAST annotates V, D, J regions and delineates framework and CDR regions using the BLAST search algorithm. Unlike other tools, IgBLAST is able to analyze nucleotide and protein sequences. The user can align to germline sequences in IMGT or other germline databases (e.g., NCBI) simultaneously for a thorough alignment. For framework and CDR delineation, the IMGT numbering scheme is used (Ye *et al.*, 2013). It is able to annotate to the allele level. This tool comes as a standalone platform or a web-based tool. As a web-based tool it allows annotation to germlines genes of five species: human, mouse, rat, rabbit and Rhesus monkey and various V, D, and J gene databases. For the standalone version of IgBLAST, there is no built-in germline reference, instead the user is required to download and build the reference database as part of the standalone installation. To do this we used the Immcantation scripts (https://bitbucket.org/kleinstein/immcantation/src/tip/scripts). These scripts download the germline reference database from IMGT and build the database in the format required by IgBLAST.

IMGT/HighV-QUEST. IMGT/HighV-QUEST was developed by the ImMunoGeneTics information system (IMGT) for the analysis of larger (≤500,000 sequences) immunoglobulin and T-cell receptor datasets, which are obtained from high-throughput sequencing.The IMGT/HighV-QUEST tool first aligns the V, J and D regions in sequential order and uses the IMGT numbering scheme. The user can select among twenty-five species such as *Homo sapiens, Mus musculus, Bos taurus, Canis lupus familiaris,* etc. (Alamyar *et al.*, 2012; Brochet *et al.*, 2008). IMGT/HighV-QUEST provides four different germline choices for *Homo sapiens* which are provided by IMGT: i) F+ORF+in-frame P (functional and ORF genes and in-frame pseudogenes); default, ii) F+ORF (only functional and ORF genes), iii) F+ORF including orphons (only functional and ORF genes including orphons), and iv) F+ORF+in-frame P including orphons (functional and ORF genes and in-frame pseudogenes including orphons). These analyses were conducted with version 3.4.15.

Abstar. Abstar is a tool that comes as part of the ab[x] package of tools for antibody NGS sequence analysis (Briney, 2018) and it performs  germline gene assignment and primary sequence annotation. The program aligns the V, D and J regions to a built-in default germline sequences of human, macaque and mice. This germline database is originated from IMGT but bundled inside the application. The algorithm used by abstar is an iterative algorithm based on combinations of BLAST and Smith-Waterman local alignments. It uses the IMGT numbering scheme for region delineations.

**Supplemental Figures**

**Supplemental Figure 1: Number and type of germline changes since the previous release**. The x-axis is the number of weeks since the previous release a new change was made. The y-axis shows the total number of changes that occurred. The red dots indicate the mean of number of changes and weeks since previous change. Red dot, gene removed; yellowish-brown, genes added; green square, alleles added; blue plus sign, sequence was changed and pink boxed-x, metadata change. The light grey box denotes the 95^th^ percentile of number of changes and number of weeks between changes.
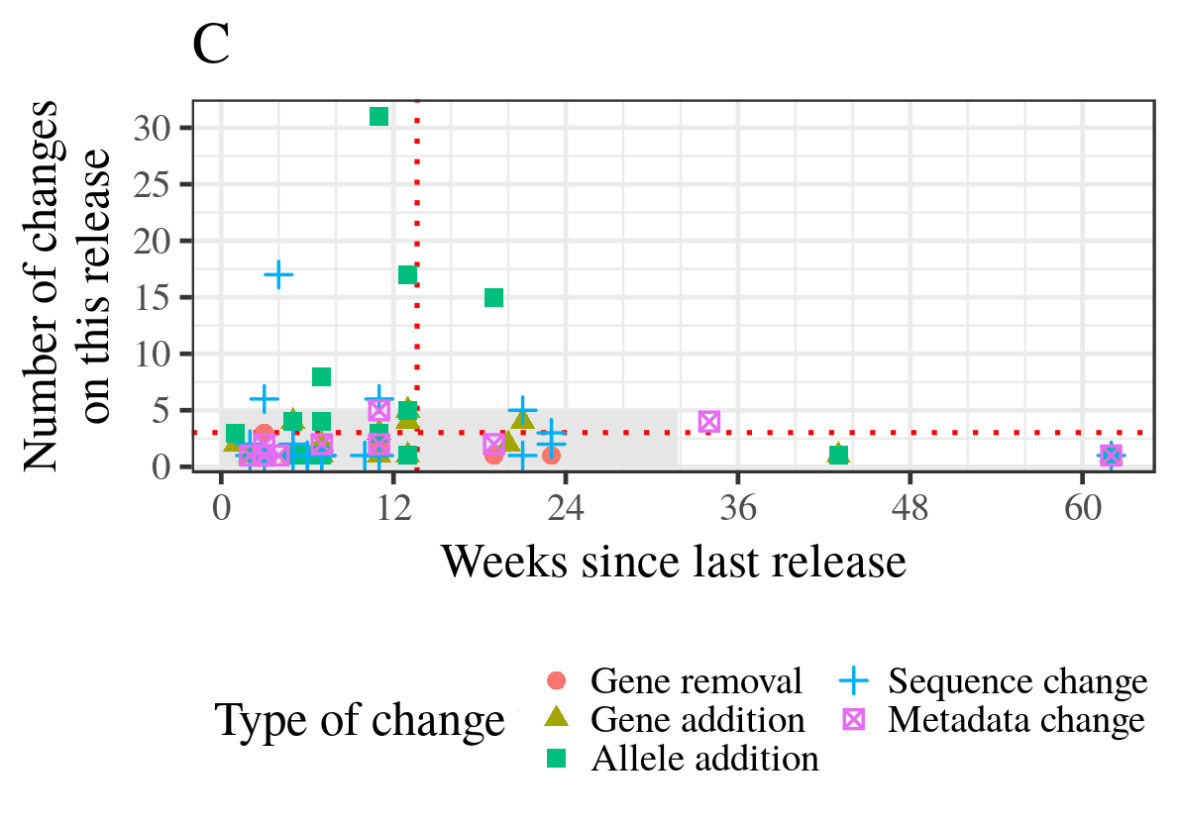


**Supplementary Figure 2:** The heat maps show the frequency of mishits of specific genes to other genes for polarized and diverse datasets by each tool. Yellow, high frequency; purple, low frequency.


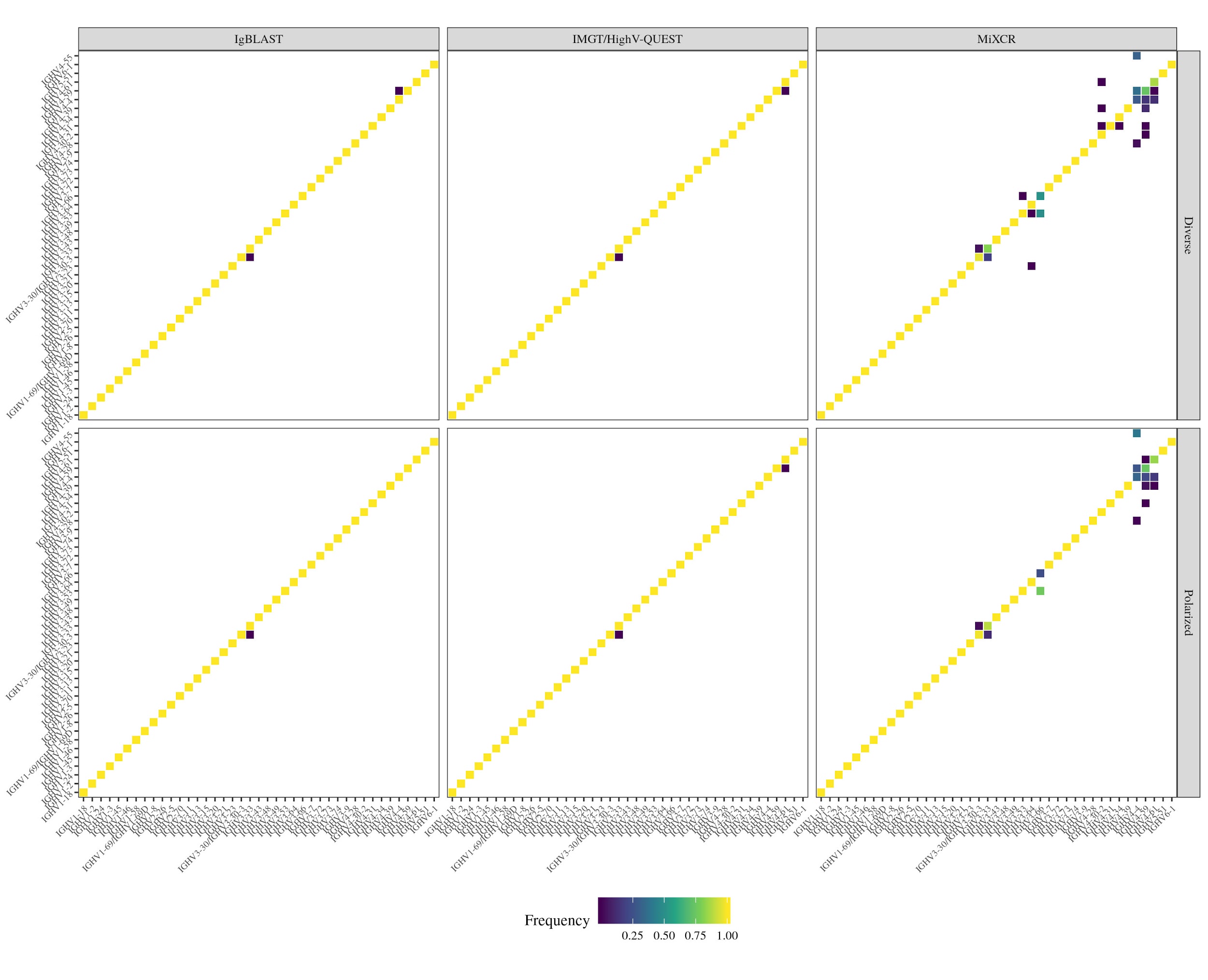


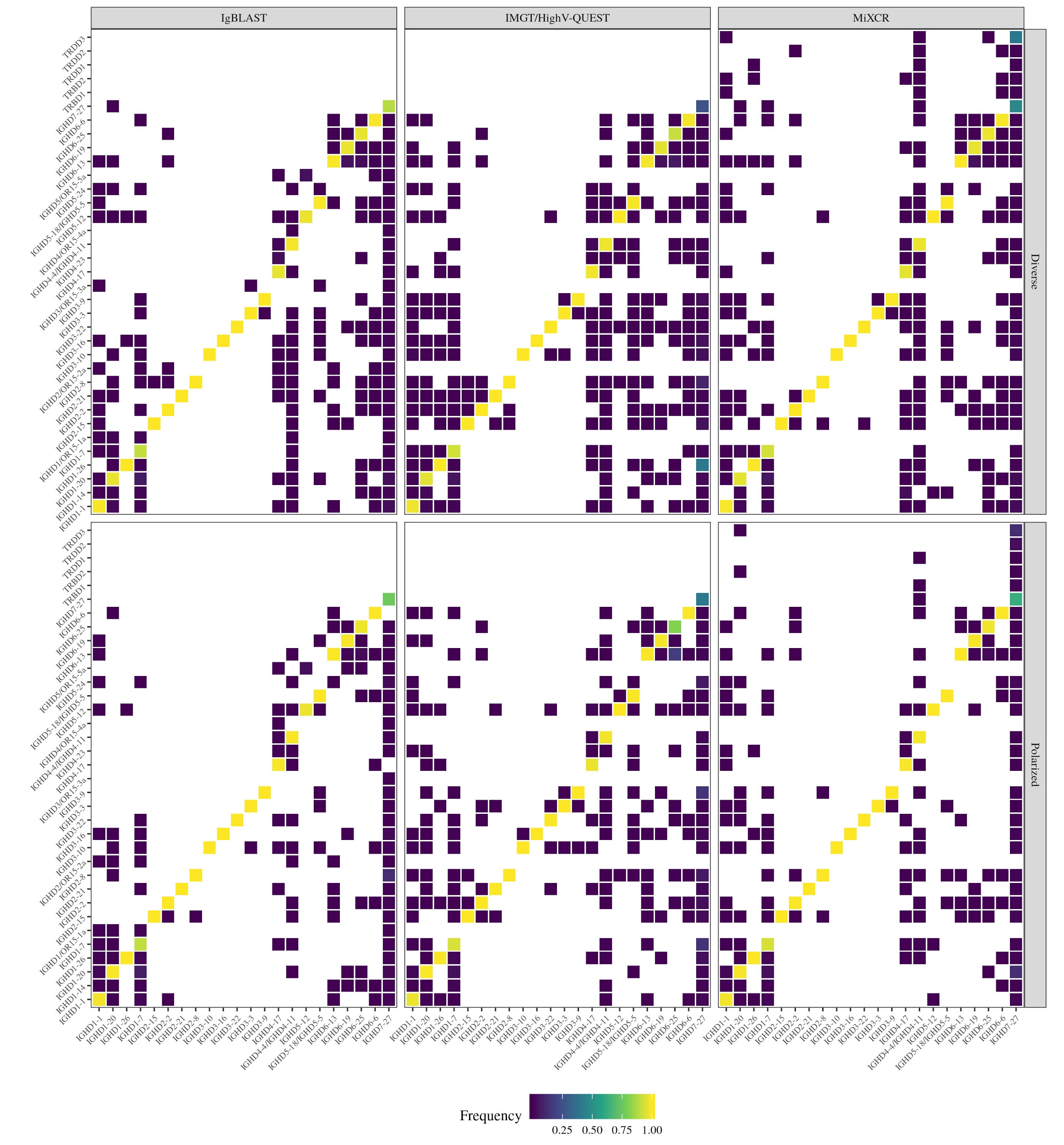


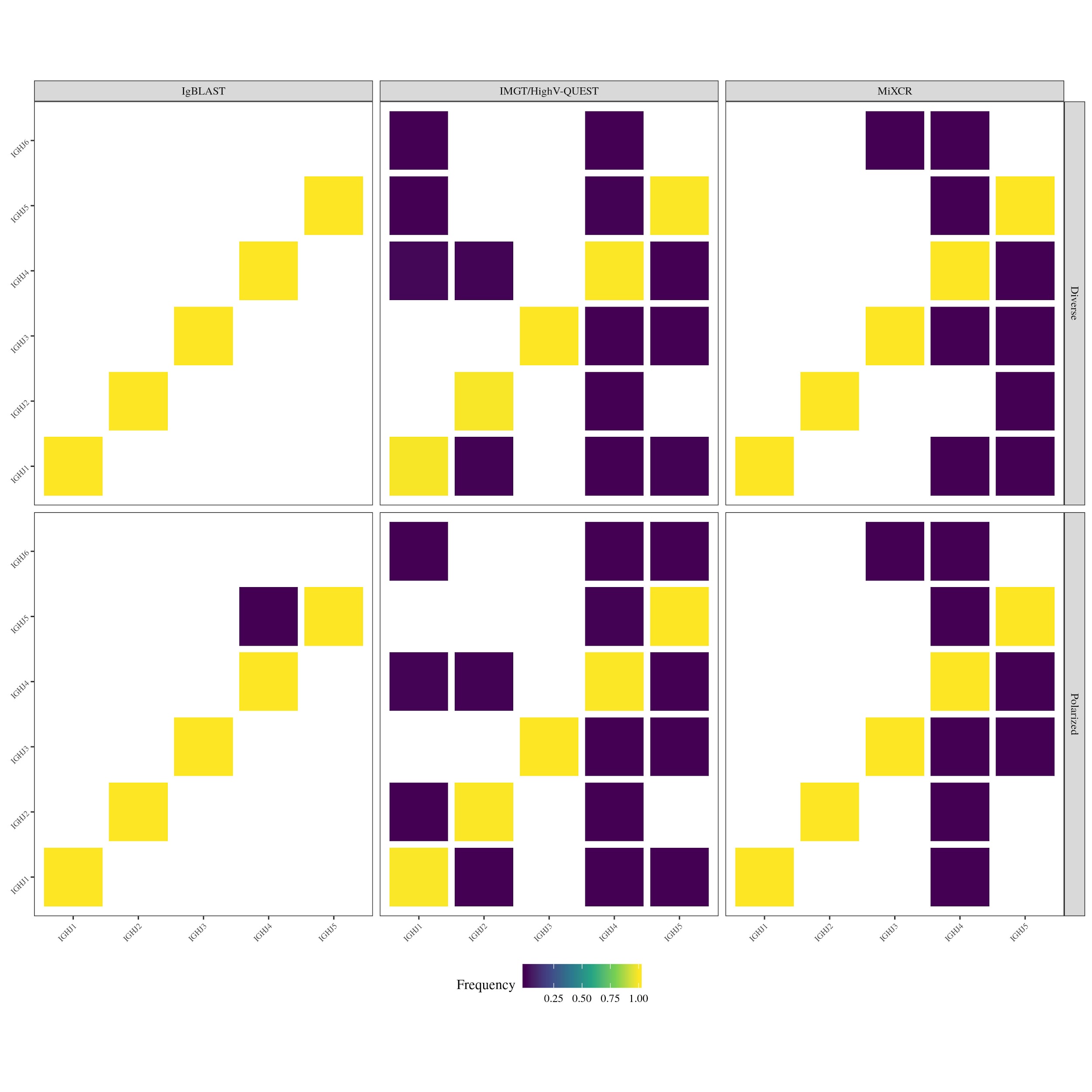


**Supplementary Figure 3: Alignment of IGHV3-66 and IGHV3-53 using Blastp Suite**.

An alignment of IGHV3-66 and IGHV3-53 was performed using Blastp suite to compare the sequence similarities between the two genes. The genes were incorrectly annotated by MiXCR but correctly annotated by IMGT/HighV-QUEST and IgBLAST demonstrating that there are differences in how these tools annotate genes.

Identities: 112/116 (97%) Positives: 113/116 (97%)

IGHV3-66 MEFGLSWVFLVAILKGVQCEVQLVESGGGLIQPGGSLRLSCAASGFTVSSNYMSWVRQAPGKGLEWVSVIYSCGSTYY

MEF LSWVFLVAI KGVQCEVQLVE+GGGLIQPGGSLRLSCAASGFTVSSNYMSWVRQAPGKGLEWVSVIYS GSTYY

IGHV3-53 MEFWLSWVFLVAISKGVQCEVQLVETGGGLIQPGGSLRLSCAASGFTVSSNYMSWVRQAPGKGLEWVSVIYSGGSTYY

IGHV3-66 ADSVKGRFTISRDNSKNTLYLQMNSLRAEDTAVYYCAR

ADSVKGRFTISRDNSKNTLYLQMNSLRAEDTAVYYCAR

IGHV3-53 ADSVKGRFTISRDNSKNTLYLQMNSLRAEDTAVYYCAR

**Supplementary Tables**

Supplementary Table 1:Benchmarked tools for antibody repertoire analysis

| Tools | IMGT/HighV-QUEST | IgBLAST | MiXCR | IgSimulator |
| --- | --- | --- | --- | --- |
| Version | 1.6.4 | 1.10.0 | 3.0 | 2.0 alpha |
| Year published | 2010 | 2013 | 2015 | 2015 |
| Summary | Analysis of B- and T- cell receptors; aligns and annotates V, D and J genes; performs junction analysis and characterizes the mutations of the variable region | Analysis of B- and T- receptors; aligns and annotates VDJ region; CDR3 identification; mutation analysis in variable region; allows user defined germline databases | Alignment, annotation and clonotyping of B- and T-cell receptor; PCR error correction, merging of paired-end reads, mutation analysis in variable region | Generates antibody repertoire sequences that mimic natural repertoire by randomly recombining V, D and J genes, inserting N, P nucleotides at the junction sites, somatic hypermutations and sequencing errors |
| Website | http://www.imgt.org/HighV-QUEST | https://www.ncbi.nlm.nih.gov/igblast/ | https://milaboratory.com/software/mixcr | https://github.com/ablab/y-tools/blob/master/ig_simulator_manual.html |
| Distribution | Web | Stand-alone/Web | Stand-alone | Web |
| Alignment algorithm | Smith-Waterman | Blast | Kaligner  Kaligner2 | NA |
| Linux installation & external tools | Web-based | C++ | Java | C++ |
| Large data sets  (> 500,000 reads) | No | Yes | Yes | Yes |
| License | Restricted for commercial use | No restrictions | Restricted for commercial use | No restrictions |
| Cited | 762 | 315 | 177 | 12 |

*Cited based on scopus.com search results on 04/30/2019

NA = not applicable

<https://www.scopus.com>

**Supplementary Table 2: Features of Immunoinformatic Tools**

| **Feature** | **IMGT/HighV-QUEST** | **IgBLAST** | **MiXCR** |
| --- | --- | --- | --- |
| Multifunctional: BCR/TCR analysis | Yes | Yes | Yes |
| Germline Reference:  Built in/User-provided | Built-in | User-provided | Both (built in: mouse, human, rat; user can provide other IMGT germline species or customized germline) |
| Assignment: Gene/Allele | Allele | Allele | Gene |
| Algorithm choice:  Built in/User-choice | Built in | Built in | User-Choice |
| Clonotyping | No | No | Yes |
| Speed | + | + | +++ |
| Computational Experience | Minimal | Advanced | Advanced |

**Supplementary Table 3: *In silico* data sets**

| Sequence composition | Target no. of reads | No. of base sequences | No. of mutated sequences | # of reads generated |
| --- | --- | --- | --- | --- |
| Diverse | 1,000,000 | 100,000 | 200,000 | 913,292 |
| Polarized | 1,000,000 | 20,000 | 100,000 | 1,067,443 |

**Supplementary Table 4: Experimental Data Sets**

| **Dataset** | **SRR no.** | **No. of reads** | **Sequencer used** |
| --- | --- | --- | --- |
| DeKosky^a^ | SRR611538 | 694,803 | Illumina MiSeq |
| Gao^b^ | SRR942698 | 294,911 | Ion Torrent |
| Zhu^c^ | SRR924017 | 460,706 | Roche 454 GS FLX |
| Heiden^d^ | SRR4026019 | 749,505 | Illumina MiSeq |

a DeKosky *et al.*, 2013; b Gao *et al.*, 2013; c Zhu *et al.*, 2013; d Heiden *et al.*, 2017

**Supplementary Table 5: IgSimulator Germline Datasets**

IGHV1-18*01

IGHV1-18*03

IGHV1-18*04

IGHV1-2*01

IGHV1-2*02

IGHV1-2*03

IGHV1-2*04

IGHV1-2*05

IGHV1-24*01

IGHV1-3*01

IGHV1-3*02

IGHV1-45*01

IGHV1-45*02

IGHV1-46*01

IGHV1-46*02

IGHV1-46*03

IGHV1-58*01

IGHV1-58*02

IGHV1-69*01

IGHV1-69*02

IGHV1-69*04

IGHV1-69*05

IGHV1-69*06

IGHV1-69*08

IGHV1-69*09

IGHV1-69*10

IGHV1-69*11

IGHV1-69*12

IGHV1-69*14

IGHV1-69D*01

IGHV1-8*01

IGHV1-8*02

IGHV1-f*01

IGHV2-26*01

IGHV2-5*01

IGHV2-5*04

IGHV6-1*01

IGHV5-a*01

IGHV5-a*02

IGHV5-a*03

IGHV5-a*04

IGHV6-1*02

IGHV7-4-1*01

IGHV7-4-1*02

IGHV7-4-1*04

IGHV7-4-1*05

IGHV2-5*05

IGHV2-5*06

IGHV2-5*07

IGHV2-5*08

IGHV2-5*09

IGHV2-5*10

IGHV2-70*01

IGHV2-70*10

IGHV2-70*11

IGHV2-70*12

IGHV2-70*13

IGHV2-70D*04

IGHV2-70D*14

IGHV3-11*01

IGHV3-11*03

IGHV3-11*04

IGHV3-11*05

IGHV3-11*06

IGHV3-13*01

IGHV3-13*02

IGHV3-13*03

IGHV3-13*04

IGHV3-13*05

IGHV3-15*01

IGHV3-15*02

IGHV3-15*03

IGHV3-15*04

IGHV3-15*05

IGHV3-15*06

IGHV3-15*07

IGHV3-15*08

IGHV3-20*01

IGHV3-21*01

IGHV3-21*02

IGHV3-21*03

IGHV3-21*04

IGHV3-23*01

IGHV3-23*02

IGHV3-23*03

IGHV3-23*04

IGHV3-30*01

IGHV3-30*02

IGHV3-30*03

IGHV3-30*04

IGHV3-30*05

IGHV3-30*06

IGHV3-30*07

IGHV3-30*08

IGHV3-30*09

IGHV3-30*10

IGHV3-30*11

IGHV3-30*12

IGHV3-30*13

IGHV3-30*14

IGHV3-30*15

IGHV3-30*16

IGHV3-30*17

IGHV3-30*18

IGHV3-30*19

IGHV3-30-3*01

IGHV3-30-3*02

IGHV3-30-3*03

IGHV3-33*01

IGHV3-33*02

IGHV3-33*03

IGHV3-33*04

IGHV3-33*05

IGHV3-33*06

IGHV3-38*03

IGHV3-43*01

IGHV3-43*02

IGHV3-43D*01

IGHV3-48*01

IGHV3-48*02

IGHV3-48*03

IGHV3-48*04

IGHV3-49*01

IGHV3-49*02

IGHV3-49*03

IGHV3-49*04

IGHV3-49*05

IGHV3-53*01

IGHV3-53*02

IGHV3-53*03

IGHV3-53*04

IGHV3-64*01

IGHV3-64*02

IGHV3-64*03

IGHV3-64*04

IGHV3-64*05

IGHV3-64D*06

IGHV3-66*01

IGHV3-66*02

IGHV3-66*03

IGHV3-66*04

IGHV3-7*01

IGHV3-7*02

IGHV3-7*03

IGHV3-72*01

IGHV3-73*01

IGHV3-73*02

IGHV3-74*01

IGHV3-74*02

IGHV3-74*03

IGHV3-9*01

IGHV3-9*02

IGHV3-9*03

IGHV3-NL1*01

IGHV3-d*01

IGHV4-28*01

IGHV4-28*02

IGHV4-28*03

IGHV4-28*04

IGHV4-28*05

IGHV4-28*06

IGHV4-28*07

IGHV4-30-2*01

IGHV4-30-2*02

IGHV4-30-2*03

IGHV4-30-2*05

IGHV4-30-2*06

IGHV4-30-4*01

IGHV4-30-4*02

IGHV4-30-4*07

IGHV4-31*01

IGHV4-39*06

IGHV4-31*02

IGHV4-31*03

IGHV4-31*04

IGHV4-31*05

IGHV4-31*10

IGHV4-34*01

IGHV4-34*02

IGHV4-34*04

IGHV4-34*05

IGHV4-34*08

IGHV4-34*09

IGHV4-34*10

IGHV4-34*11

IGHV4-34*12

IGHV4-38-2*01

IGHV4-38-2*02

IGHV4-39*01

IGHV4-39*02

IGHV4-39*05

IGHV4-39*07

IGHV4-4*01

IGHV4-4*02

IGHV4-4*07

IGHV4-4*08

IGHV4-59*01

IGHV4-59*02

IGHV4-59*03

IGHV4-59*04

IGHV4-59*05

IGHV4-59*06

IGHV4-59*07

IGHV4-59*08

IGHV4-59*10

IGHV4-61*01

IGHV4-61*02

IGHV4-61*03

IGHV4-61*05

IGHV4-61*08

IGHV4-b*01

IGHV4-b*02

IGHV5-51*01

IGHV5-51*02

IGHD1-1*01

IGHD1-20*01

IGHD1-26*01

IGHD1-7*01

IGHD2-15*01

IGHD2-2*01

IGHD2-2*02

IGHD2-2*03

IGHD2-21*01

IGHD2-21*02

IGHD2-8*01

IGHD2-8*02

IGHD3-10*01

IGHD3-10*02

IGHD3-16*01

IGHD3-16*02

IGHD3-22*01

IGHD3-3*01

IGHD3-3*02

IGHD3-9*01

IGHD4-17*01

IGHD4-4*01

IGHD5-12*01

IGHD5-18*01

IGHD5-5*01

IGHD6-13*01

IGHD6-19*01

IGHD6-25*01

IGHD6-6*01

IGHD7-27*01

IGHJ1*01

IGHJ2*01

IGHJ3*01

IGHJ3*02

IGHJ4*01

IGHJ4*02

IGHJ4*03

IGHJ5*01

IGHJ5*02

IGHJ6*01

IGHJ6*02

IGHJ6*03

IGHJ6*04

IGHV5-51*03

IGHV5-51*04

**Supplementary Table 6: List of changes to the IMGT germline for the last 10 years**

| **Release #** | **gene removals** | **gene additions** | **allele additions** | **sequence changes** | **metadata changes** |
| --- | --- | --- | --- | --- | --- |
| *201914-2* |  | 1 | 4 | 1 |  |
| *201907-2* | 1 |  |  |  | 2 |
| *201851-4* |  |  |  | 1 |  |
| *201845-4* |  | 1 | 1 |  |  |
| *201839-3* |  | 1 | 1 | 1 | 1 |
| *201836-1* |  | 4 | 4 | 2 |  |
| *201830-3* |  | 2 | 3 |  |  |
| *201829-3* |  | 1 | 1 | 1 |  |
| *201824-3* |  |  |  | 1 |  |
| *201814-3* |  | 2 | 8 |  |  |
| *201806-4* |  |  | 1 |  |  |
| *201751-3* |  |  |  | 2 |  |
| *201728-2* |  |  |  |  | 1 |
| *201723-4* |  |  |  |  | 4 |
| *201640-5* | 1 | 1 | 1 | 1 | 1 |
| *201531-2* |  | 1 |  | 1 | 1 |
| *201528-4* |  | 4 | 5 |  |  |
| *201515-3* | 3 |  |  |  | 2 |
| *201511-4* |  |  |  | 6 | 1 |
| *201508-2* |  |  |  | 17 |  |
| *201504-1* | 1 |  | 15 |  | 2 |
| *201436-3* |  | 4 |  | 5 |  |
| *201414-4* |  |  |  |  | 1 |
| *201410-5* |  | 1 |  | 2 |  |
| *201408-4* |  | 3 | 3 | 1 | 2 |
| *201349-1* | 2 |  |  | 6 | 5 |
| *201338-1* |  | 1 | 1 |  |  |
| *201325-1* |  | 5 | 17 |  |  |
| *201310-4* |  | 1 | 1 |  |  |
| *201308-3* | 1 |  |  | 3 |  |
| *201237-3* |  |  |  | 1 |  |
| *201230-5* |  |  |  | 1 |  |
| *201209-4* |  |  |  | 1 |  |
| *201201-5* |  | 1 | 1 |  |  |
| *201110-1* |  | 1 | 31 |  |  |
| *201051-1* |  | 2 |  |  |  |
| *201030-3* | 0 | 0 | 0 | 0 | 0 |

**Supplementary Table 7: List of duplicate sequences in IgSimulator default germline database**

| Genes | | Sequence |
| --- | --- | --- |
| IGHV4-38-2 | IGHV4-b | caggtgcagctgcaggagtcgggcccaggactggtgaagccttcggagaccctgtccctcacctgcactgtctctggttactccatcagcagtggttactactggggctggatccggcagcccccagggaaggggctggagtggattgggagtatctatcatagtgggagcacctactacaacccgtccctcaagagtcgagtcaccatatcagtagacacgtccaagaaccagttctccctgaagctgagctctgtgaccgccgcagacacggccgtgtattactgtgcgaga |
| IGHV1-69 | IGHV1-69D | caggtgcagctggtgcagtctggggctgaggtgaagaagcctgggtcctcggtgaaggtctcctgcaaggcttctggaggcaccttcagcagctatgctatcagctgggtgcgacaggcccctggacaagggcttgagtggatgggagggatcatccctatctttggtacagcaaactacgcacagaagttccagggcagagtcacgattaccgcggacgaatccacgagcacagcctacatggagctgagcagcctgagatctgaggacacggccgtgtattactgtgcgagaga |
| IGHV3-30 | IGHV3-30-3 | caggtgcagctggtggagtctgggggaggcgtggtccagcctgggaggtccctgagactctcctgtgcagcctctggattcaccttcagtagctatgctatgcactgggtccgccaggctccaggcaaggggctggagtgggtggcagttatatcatatgatggaagtaataaatactacgcagactccgtgaagggccgattcaccatctccagagacaattccaagaacacgctgtatctgcaaatgaacagcctgagagctgaggacacggctgtgtattactgtgcgagaga |
| IGHD5-18 | IGHD5-5 | gtggatacagctatggttac |

**Supplementary Table 8: Non-overlapping CDR3s are in part a result of algorithm and alignment differences between tools.**

|  | | IgBLAST | IMGT/HighV-QUEST |
| --- | --- | --- | --- |
| Total no. of input sequences annotated | | 759,505 | 759,505 |
| Total no. of input sequences whose CDR3's do not overlap | | 51,791  (6.8%) | 14,844  (1.95%) |
|  | Total no. of input sequences identified by both tools | 8,983  (1.2%) | |
|  | Total no. that have same VDJ but different annotation | 7,735  (1.0%) | |
|  | Total no. that have different VDJ and different annotation | 1,248  (0.2%) | |

**Supplementary Table 9: Other Immuno-informatic tools for B- & T-cell Receptor Repertoire Analysis**

| Tools | Cell type | Year | Summary |  | Website |
| --- | --- | --- | --- | --- | --- |
| AbMining ToolBox | B cell | 2013 | Package for analysis of antibody libraries sequenced by 454, Ion Torrent, MiSeq sequencers; Heavy chain CDR3 identification |  | https://sourceforge.net/projects/abmining/ |
| Absim | B cell | 2016 | simulates the stages between antibody sequence evolution and it gives the possibility to modeling immunologically relevant parameters e.g. period of repertoire evolution or the frequency of the mutations |  | https://CRAN.R-project.org/package=AbSim |
| Immcantation/alakazam | B cell | 2015 | clonal lineage reconstruction, lineage topology analysis, repertoire diversity, V(D)J gene usage and physicochemical property analysis |  | https://alakazam.readthedocs.io/en/version-0.2.11/ |
| Immcantation/BASELINe | B cell | 2012 | Bayesian estimation of antigen driven selection based on analysis of somatic mutation patterns and quantifying the selection The most up-to-date version of the method is available in the R package SHazaM |  | http://selection.med.yale.edu/baseline/ |
| Immcantation/Change-O | B cell | 2015 | Toolkit; VDJ reference alignment standardization, germline reconstruction, clonotyping, creating lineage trees, inferring somatic hypermutation targeting models, measuring repertoire diversity, quantifying selection pressure and calculate sequence chemical properties |  | https://immcantation.readthedocs.io/en/version-2.7.0/ |
| HTJoinSolver | B cell | 2015 | Identification of V and J genes with insertions and deletions (indels) in mutated sequences |  | https://dcb.cit.nih.gov/HTJoinSolver |
| IgAT | B cell | 2012 | An Excel based tool for IMGT/HighV-QUEST output files to generate descriptive statistics and figures of gene usage, length and composition of the CDR-3 region; calculate the probability of antigen selection based on somatic mutational patterns, the average hydrophobicity of the antigen-binding sites, and predictable structural properties of the CDR-H3 loop |  | www.uni-marburg.de/neonat/igat |
| IgDiscover | B cell | 2016 | Identifies germline V genes from expressed antibody repertoires |  | https://pypi.org/project/igdiscover |
| IGGalaxy | B cell | 2014 | Web based application for identifying IGH gene rearrangements for both repertoire and clonality studies; multi-sample and multi-replicate input analysis for both IgBLAST and IMGT/HighV-QUEST. |  | http://bioinformatics.erasmusmc.nl/wiki/index.php/Immunoglobulin_Galaxy |
| IgRepertoireConstructor | B cell | 2015 | Toolkit that performs error-correction; uses mass spectra to validate the constructed repertoire; clustering and computing consensus sequence and abundance. |  | https://yana-safonova.github.io/ig_repertoire_constructor/ |
| IgSCUEAL | B cell | 2013 | V, J alignment and annotation based on phylogenetic trees; gene usage; CDR3 length |  | http://hyphy.org/w/index.php/IgSCUEAL |
| iHMMune-align | B cell | 2007 | Model processes involved in IgH gene rearrangement and maturation using HMM; identifying germline genes of IgH to generate an alignment |  | https://cgi.cse.unsw.edu.au/~ihmmune/iHMMune/ |
| ImmunediveRsity | B cell | 2015 | quality filtering, sequencing noise correction and V, D, J alignments, clonotyping |  | https://bitbucket.org/ImmunediveRsity/immunediversity |
| sciReptor | B cell | 2015 | Toolkit for processing and analysis of antigen receptor repertoire sequencing data at single-cell level |  | https://github.com/b-cell-immunology/sciReptor |
| SONAR | B cell | 2016 | SONAR creates annotation of germline V(D)J genes and lineage identification using longitudinal NGS samples |  | https://github.com/scharch/SONAR |
| Immcantation/TIgGER | B cell | 2015 | Detect novel alleles; novel polymorphism detection; genotyping |  | https://tigger.readthedocs.io/en/0.3.1/ |
| TRIgS | B cell | 2015 | Toolkit used for quality control, junction analysis, clonal analysis, phylogenetic analysis of selected sugroups, germline analysis and CDR3 length distribution analysis |  | http://cimm.ismb.lon.ac.uk/trigs/ |
| VDJFasta | B cell | 2011 | Alignment, CDR3 extraction using a hidden Markov model (HMM) |  | https://sourceforge.net/projects/vdjfasta/ |
| VDJSolver | B cell | 2015 | V, J gene alignment using hidden Markov model or maximum likelihood |  | http://www.cbs.dtu.dk/services/VDJsolver/ |
| Vidjil | B cell | 2013-2014 | Extracts CDR3s with the overlapping VDJ junction; then compute a representative sequence for each clone |  | http://www.vidjil.org |
| Antigen Receptor Galaxy | Both | 2017 | Toolkit for demulitiplexing, annotation with IMGT/HighV-QUEST, gene usage, clonailty, CDR features, diversity |  | https://bioinf-galaxian.erasmusmc.nl/argalaxy/ |
| sumrep | Both | 2019 | Summarizing, visualizing, and comparing immune receptor repertoires |  | https://github.com/matsengrp/sumrep |
| bcRep | Both | 2016 | Analyze IMGT/HighV-QUEST data; functionality, junction frames, gene usage, mutations, statistics, clonality; visualization |  | https://cran.r-project.org/web/packages/bcRep/vignettes/vignette.html |
| BRepertoire | Both | 2018 | Web based tool for repertoire statistical analysis; gene using, physico-chemical properties of CDR regions and clonotyping |  | http://mabra.biomed.kcl.ac.uk/BRepertoire |
| ClonoCalc*Plot | Both | 2017 | Generates descriptive and comparative statistical analysis for visualization |  | https://bitbucket.org/ClonoSuite/clonocalc-plot |
| IGoR | Both | 2018 | Calculates VDJ recombination and somatic hypermutation statistics |  | https://github.com/qmarcou/IGoR |
| IMEX | Both | 2015 | statistical analysis; CDR and V(D)J analysis; diversity analysis; primer efficiency and comparison of multiple datasets; uses IMGT/HighV-Quest analysis outputs |  | http://bioinformatics.fh-hagenberg.at/immunexplorer/ |
| partis | Both | 2016 | B- and T-cell receptor sequence annotation, simulation, clonal family and germline inference |  | https://github.com/psathyrella/partis/blob/master/manual.md |
| immuneDB | Both | 2015 | V and J gene identification, clonotyping, lineage construction, selection pressure calculation |  | https://immunedb.readthedocs.io |
| IMonitor | Both | 2015 | Uses realignment to identify V(D)J genes and alleles after common local alignment; corrects the PCR and sequencing errors and minimizes PCR bias of rearranged sequences with different V and J gene subgroups; statistics, visualizations |  | https://github.com/zhangwei2015 |
| IMPre | Both | 2016 | Prediction of T- and B-cell receptor germline genes and alleles from repertoire data |  | https://github.com/zhangwei2015/IMPre |
| IMSEQ | Both | 2015 | Fast, PCR and sequencing error identification tool from single or paired-end data; annotation, clonotyping |  | http://www.imtools.org |
| LymAnalyzer | Both | 2016 | VDJ gene alignment; CDR3 extraction; polymorphism analysis; lineage mutation tree construction |  | https://sourceforge.net/projects/lymanalyzer/ |
| LymphoSeq | Both | 2015 | Analyzes TCRs and BCRs CDR3 sequences generated by Adaptive Biotechnologies' ImmunoSEQ assay, MiXCR, IMGT/HighV-Quest (annotation; clonotyping |  | https://bioconductor.org/packages/release/bioc/html/LymphoSeq.html |
| MaxSnippetModel | Both | 2017 | Diagnose disease from immune repertoires |  | https://github.com/jostmey/MaxSnippetModel |
| MiGEC | Both* | 2013 | Filters out erroneous PCR and sequencing errors from sequencing data generated with molecular barcoding; hotspot error correction algorithm; can be used in combination with MiXCR; only produces clonotypes as V+D+J+CDR3 set and does not account for hypermutations |  | https://milaboratory.com/software/migec |
| Immcantation/pRESTO | Both | 2013 | a toolkit that processes sequences before germline gene assignment; single reads or paired-end reads; quality control, primer masking, annotation of reads with sequence embedded barcodes, generation of unique molecular identifier (UMI) consensus sequences, assembly of paired-end reads and identification of duplicate sequences |  | https://presto.readthedocs.io |
| RDI | Both | 2016 | Quantifies differences in V, D, J gene usage between repertoires |  | https://bitbucket.org/cbolen1/rdicore |
| Recon | Both |  | Measures repertoire diversity |  | http://arnaoutlab.github.io/Recon |
| repgenHMM | Both | 2016 | Generate synthetic sequences, calculate the probability of generation of any receptor sequence, and measure theoretical diversity of the repertoire |  | https://bitbucket.org/yuvalel/repgenhmm |
| TRIg | Both | 2016 | Handles non-regular T cell receptor and Ig sequences; alignments to the whole receptor gene instead of gene. |  | https://github.com/TLlab/trig |
| VDJ | Both | 2014 | Alignment, annotation, annotate isotypes, clustering CDR3 |  | https://github.com/laserson/vdj |
| VDJMLpy | Both | 2016 | Python module for working with the results of immune receptor sequence alignment in VDJML format. |  | https://vdjserver.org/vdjml/ |
| VDJPuzzle | Both | 2016 | Construct TCRs and BCRs from single cell RNA sequencing data |  | https://github.com/simone-rizzetto/VDJPuzzle |
| VDJServer | Both | 2014-2018 | Preprocessing, quality control, VDJ gene assignment, repertoire characterization and repertoire comparison visualizations; public sharing of repertoire sequencing data |  | https://vdjserver.org/docs/index.html |
| VDJviz | Both* | 2015 | A web-based graphical user interface used to visualize results from MiTCR, MiXCR, MiGEC and MiGMAP RepSeq; clonotype table browsing with VDJ regions, CDR3 pattern matching for a single sample and across multiple samples, currently does not account for somatic hypermutations |  | https://github.com/antigenomics/vdjviz |
| ALPHABETR | T cell | 2016 | Pairs alpha and beta TCRs; CDR3A/CDR3B extraction |  | https://github.com/edwardslee/alphabetr |
| clonotypeR | T cell | 2013 | Identify and analyze clonotypes from high-throughput T cell receptors sequence libraries and prepare these sequences for differential expression analysis |  | http://clonotyper.branchable.com |
| Decombinator | T cell | 2013 | TCR repertoire anaylsis produced by deep sequencing; error correction; fast string matching algorithm; CDR3 extraction; large datasets >500’000 reads. |  | https://github.com/innate2adaptive/Decombinator |
| MiTCR | T cell | 2013 | CDR3 extraction, identify V, D, J genes, clonotyping, filters or rescues low quality reads, PCR and sequencing error correction; analysis large datasets (>500’000 reads). |  | https://github.com/milaboratory/mitcr |
| RTCR | T cell | 2016 | PCR and sequencing error correction; alignment, annotation; CDR3 extraction |  | https://github.com/uubram/RTCR |
| tcR | T cell | 2015 | Diversity measures, shared TCR sequence identification, gene usage statistics |  | http://imminfo.github.io/tcr/ |
| TCRklass | T cell | 2015 | Alignment, annotation, clonotyping |  | http://sourceforge.net/projects/tcrklass |
| TraCeR | T cell | 2015 | Reconstruct full-length, paired TCR sequences from single-cell T lymphocyte RNA sequence data; clonotyping |  | https://github.com/Teichlab/tra[cer](https://github.com/Teichlab/tracer) |
| VDJtools | Both | 2015 | Post-analysis of TCR repertoire sequencing data; computes statistics; perform cross-sample analysis; clonotype table filtering; repertoire clustering; clonotype table joining; does not account for hypermutations |  | https://github.com/mikessh/vdjtools |

**Supplementary Table 9**

**Command lines or parameter descriptions**

**MiXCR**

to align to default germline

mixcr align --species hs -r input.fasta -p kAligner2

to export raw alignments

mixcr exportAlignments -descrsR1 -vHits -dHits -jHits -chains

align.output.vdjca alignments.txt

to compile cdr3 clones

mixcr assemble align.output.vdjca clones.clns

to export raw CDR3 clones

mixcr exportClones -count -vHit -dHit -jHit -aaFeature CDR3 clones.clns clones.txt

to export "preprocessed" CDR3 clones

mixcr exportClones -count -vHit -dHit -jHit -aaFeature CDR3 --filter-out-of-frames --filter-stops  clones.clns clones.txt (further processed manually according to description in methods)

**IgBLAST**

AssignGenes.py igblast -s input.fasta -b referece.germline --organism human --loci ig --format air

AssignGenes.py is part of the Imcanntation repo scripts, and is a wrapper to simplify the otherwise longer igblast command.

**IMGT/HighV-QUEST**

Species: Homo sapiens (human)

Receptor type or locus: IGH

Reference dictionary is set to F+ORF+in-frame P (default)

Alignment for D-GENE

Other parameters are default

**IgSimulator**

ig_simulator.py --chain-type HC --num-bases $1 --num-mutated $2 --repertoire-size 1000000 --vgenes IGHV.fasta --dgenes IGHD.fasta --jgenes IGHJ.fasta --db-type imgt -o name.output

diverse: $1 = 100,000 $2 = 200,000

polarized: $1 = 20,000 $2 = 100,000
